# Association between level of depression and coronary heart disease, stroke risk and all-cause and cardiovascular mortality: Data from the 2005–2018 National Health and Nutrition Examination Survey

**DOI:** 10.3389/fcvm.2022.954563

**Published:** 2022-10-26

**Authors:** Ruihuan Shen, Ning Zhao, Jia Wang, Peiyao Guo, Shuhui Shen, Donghao Liu, Tong Zou

**Affiliations:** ^1^Department of Cardiology, National Center of Gerontology, Peking Union Medical College, Beijing Hospital, Institute of Geriatric Medicine, Chinese Academy of Medical Sciences, Beijing, China; ^2^Department of Gastrointestinal Surgery, Department of General Surgery, National Center of Gerontology, Peking Union Medical College, Beijing Hospital, Institute of Geriatric Medicine, Chinese Academy of Medical Sciences, Beijing, China; ^3^Department of Cardiology, National Center of Gerontology, Beijing Hospital, Institute of Geriatric Medicine, Peking University Fifth School of Clinical Medicine, Beijing, China

**Keywords:** coronary heart disease, stroke, depression, NHANES, PHQ-9, mortality

## Abstract

Research on the association between level of depression and coronary heart disease (CHD), stroke risk, and all-cause and cardiovascular mortality is lacking in large-scale or population-based studies incorporating cardiovascular disease (CVD) endpoints. We aim to assess the relationship between the level of a person's depression and their risk of CHD, stroke, and all-cause and cardiovascular mortality. Utilizing data from the United States National Health and Nutrition Examination Survey (NHANES), multicycle cross-sectional design and mortality linkage studies were conducted. The study sample included 30918 participants aged 20–85 years old during the 2005–2018 period. Depression was assessed using the nine-item Patient Health Questionnaire (PHQ-9), with scores of 5, 10, 15, and 20 being the cut-off points for mild, moderate, moderately severe, and severe depression, respectively. A series of weighted logistic regression analyses and Cox proportional hazards models were utilized to examine the relationship between the level of depression with the risk of CHD, stroke, all-cause, and cardiovascular mortality. Trend analyses were conducted by entering the level of depression as a continuous variable and rerunning the corresponding regression models. Weighted logistic regression models consistently indicated a statistically significant association between the level of depression and increased risk of CHD and stroke, and those linear trend tests were statistically significant (P for trend < 0.001). Furthermore, weighted Cox regression analyses consistently indicated that participants who had a more severe degree of depression were at a higher risk of all-cause death, and trend analyses suggested similar results (P for trend < 0.001). Another weighted Cox regression analysis also consistently indicated that except for severe depression, the hazard of cardiovascular death was increased with each additional level increase of depression. Our study confirmed that the level of depression was strongly associated with CHD, stroke, and all-cause and cardiovascular mortality, even after accounting for other factors that could impact risk, including variables of age, gender, ethnicity, income, education, body mass index (BMI), marital, and smoking status.

## Introduction

According to the World Health Organization (WHO), depression was ranked as the single largest contributor to global disability and non-fatal health loss; current estimates are that approximately 4.4% of the population worldwide, that is to say, about 264 million people, suffers from depressive disorder—a chronic and recurrent condition—imposing a significant extra burden on public health (1).

Cardiovascular diseases often co-occur with depression (2–4), and the two are projected to be the top two leading contributors to the global disease burden by 2030 (5). In particular, CHD was also the main cause of global morbidity and mortality, being responsible for roughly one-third of all deaths for people aged over 35 years worldwide (6).

Cardiovascular diseases were influenced by and associated with a variety of aspects of health and wellbeing, especially mental health. A recent study has reported that depression symptom history may be a predictor or marker of cardiometabolic risk over decades (7). Thus, by understanding the impact of the link, doctors can improve a patient's overall health by addressing both mental health and heart disease together. Several prior research studies have examined the relationship between depression and CVDs from two perspectives: depression's impact on CVDs and depression as a risk factor for poor prognosis among patients with CVDs (3, 8–12).

To date, studies have demonstrated that depression is a leading contributor to an elevated risk of morbidity and mortality for CVDs, such as CHD and stroke (9, 13–15). However, until recently, research on the association between the level of depression and CHD, stroke risk, and all-cause and cardiovascular mortality are nevertheless lacking in large-scale or population-based studies incorporating CVD endpoints.

We hypothesized that the severity of a person's depression may elevate their risk of CHD, stroke, and mortality. Thus, to test our hypothesis, multicycle cross-sectional design and mortality linkage studies were conducted to assess the relationship between the level of depression and CHD, stroke risk, and mortality, utilizing data from the NHANES.

## Materials and methods

### Database

Data were collected from the multiple cycles of the United States cross-sectional Continuous NHANES from 2005 to 2018. Moreover, the National Center for Health Statistics (NCHS) at the Centers for Disease Control and Prevention (CDC) (16) provided NHANES public-use linked mortality follow-up files until 31 December 2019, and it has linked several population surveys to death certificate records from the National Death Index (NDI) (17).

The NHANES uses a complex, stratified multistage, probability cluster design to produce a nationally representative survey of the health and nutritional status of the non-institutionalized civilian population in the United States, with full details provided in the NHANES survey methods and analytic guidelines (18). Information on nutritional health and the status of non-institutionalized civilians of the United States population is gathered through a series of home interviews, examinations, and laboratory measurements.

### Study population

The original sample is comprised of seven successive cycles collected in 2-year increments from 2005 to 2018 of the continuous NHANES. The inclusion criteria were as follows: participants from the 2005 to 2018 annual NHANES cycles aged 20–85 years at the time when NHANES data were collected where there was accessible data on CHD and stroke status and where the participant depression questionnaire data were included in the analysis.

### Data collection

This is a prospective cohort study. Information on demographics, comorbidities, and body measurements was collected. Demographic and comorbidities data were recorded in a home interview by household questionnaire. Standardized body measurements (e.g., BMI) and the questions from the PHQ-9 were provided by trained field health technicians for all examinees in the mobile examination center (MEC).

### Laboratory tests

On the Modular Chemistry side of the DxC800, the concentration of blood glucose and 2-h oral glucose tolerance test (OGTT) blood glucose in biological fluids were determined by the oxygen rate method by employing a Beckman Oxygen electrode (glucose oxidase method). A precise volume of sample was introduced in a reaction cup containing an electrode that responds to oxygen concentrations. Electronic circuits determined the rate of oxygen consumption, which was directly proportional to the concentration of glucose in the sample.

Hemoglobin A1c (HbA1c) was measured in whole blood at the University of Missouri-Columbia using the Primus CLC 330 and Primus CLC 385 instruments (Primus Corporation, Kansas City, Missouri, USA) for the high-performance chromatography.

These laboratory procedure manuals were available on the NHANES website (18).

### Primary study variables

#### Assessment of CHD and stroke

Participants who answered “yes” to the question “has a doctor or other health professional ever told you that you had CHD/stroke?” on the medical conditions section of the household questionnaire through home interview were considered to have CHD/stroke.

### Independent variable

#### Assessment of depressive symptoms

The PHQ-9 represents a nine-item instrument for screening depression. And the instrument incorporates the Diagnostic and Statistical Manual (DSM)-IV depression diagnostic criteria (19).

Depression was measured using PHQ-9, a continuum scale of severity from minimal to severe, with scores of 5, 10, 15, and 20 being the cut-off points for mild, moderate, moderately severe, and severe depression, respectively. Those with PHQ-9 total scores ≥10 were considered as having clinically relevant depression (19, 20), which corresponded with moderate to severe depression symptoms.

### Covariates and confounders

A number of potential confounding variables were taken into consideration. Age was split into groups: 20–30, 31–40, 41–50, 51–60, 61–70, 71–80, and 81–85 years old. Gender was divided into male and female participants. Ethnicity was classified as White, Black, Mexican, and other races. The classification of marital status included married, living with partner, separated, divorced, widowed, and never married. Educational level was categorized as college graduate or above, some college or AA degree, high school graduate, 9–11th grade, and less than ninth grade.

The poverty income ratio (PIR) was used to measure income, which was calculated by dividing family income by the poverty guidelines, specific to family size, as well as the appropriate year and state. The values were not computed if the income screener information [income questionnaire (INQ) 220: < $20,000 or ≥ $20,000] was the only family income information reported. If family income was reported as a range value, the midpoint of the range was used to compute the variable. Values at or above 5.00 were coded as 5.00 or more due to disclosure concerns. There were five distinct categories of PIR: poor (i.e., <1.0), nearly poor (i.e., 1.0–1.9), middle income (i.e., 2.0–3.9), high income (i.e., ≥4.0), and unknown (not acquired).

Smoking status was categorized as former, current, or never. BMI was divided into four categories according to the CDC classification for adults 20 years old and older: low (<18.5 kg/m^2^), normal (18.5–25 kg/m^2^), and overweight (≥25 kg/m^2^) (21).

### History of CVDs, cancer, or malignancy

Information on past medical history was self-reported by participants. Regarding the question “Have you ever been told by a doctor or health professional that you had CHD/angina, also called angina pectoris/heart attack (also called myocardial infarction)/stroke/congestive heart failure (CHF)/cancer or a malignancy of any kind?”, persons who answered “yes” were perceived as having a history of CVDs, cancer, or malignancy.

### Other comorbid conditions

Information on comorbidities was self-reported by participants. Regarding the question “Have you ever been told by a doctor or health professional that you have …?”, persons who answered “yes” were perceived as having the following comorbidities: hypertension, CHF, angina/angina pectoris, arthritis, and hyperlipidemia.

Other than that, Parkinson's disease was diagnosed by taking anti-Parkinson agents, and the diagnostic criteria for diabetes were as follows: doctor-diagnosed diabetes; glycohemoglobin HbA1c (%) > 6.5; random blood glucose (mmol/l) ≥ 11.1; 2-h OGTT blood glucose (mmol/l) ≥ 11.1; and use of diabetes medication or insulin.

### Follow-up

The period of follow-up was from the date of the interview to the last follow-up time, 31 December 2019, or the date of death. Causes of death for these included participants were documented in death certificate records from the NDI.

### Outcomes

The endpoints for this study were all-cause and cardiovascular mortality. All-cause mortality encompassed all known and unknown causes, whereas cardiovascular mortality was defined using the International Classification of Diseases coding (ICD-9 and ICD-10), including the death from diseases of the heart (I00–I09, I11, I13, I20–I51) and cerebrovascular diseases (I60–I69).

### Statistical analysis

R software (version 4.1.2, https://www.R-project.org) was utilized to conduct statistical analysis.

In the original NHANES surveys, responses coded as “missing,” “refused,” or “do not know” were treated as missing. Participants with missing data in one of the primary study covariates mentioned above or without mortality information were not included for further analysis.

Weighted proportions of descriptive statistics were employed to summarize the characteristics of the study sample; the design-based χ^2^-test was used to examine the associations of categorical variables with depression.

A subgroup analysis was conducted to investigate whether the association between CHD, stroke, and depression varied across different subgroups of study covariates and comorbid conditions, separately. We examined the interaction effects of CHD and stroke with PHQ-9 score in several participant subgroups (gender, age grouping, ethnicity, marital status, educational level, PIR grouping, smoking status, BMI grouping), and the *P*-value for interaction was determined by the Wald test.

After univariate analysis and referring to previous studies and related literature (22–26), weighted logistic regression analyses were conducted to assess the association of CHD and stroke with the severity of depression in three models to control for potential confounding variables. Model 1 was the unadjusted model; model 2 included the level of depression, age, gender, and ethnicity; and model 3 adjusted for age, gender, ethnicity, marital status, family PIR, education, smoking status, and BMI. Crude and adjusted odds ratios (ORs) and their 95% confidence intervals (CIs) between CHD, stroke, and depression severity were presented.

Likewise, after excluding the participants with CVDs, cancer, or malignancy at baseline, a series of weighted Cox regression analyses was performed to estimate the relationships between the level of depression and risk of having all-cause and cardiovascular mortality, respectively, after adjusting for potential confounders in three models. Model 1 only included the level of depression, and no covariates were adjusted. Model 2 adjusted for age, gender, and ethnicity. Model 3 was further adjusted for marital status, family PIR, education, smoking status, and BMI. Crude and adjusted hazard ratios (HR) and their 95% CI between the level of depression and outcomes were presented.

We determined the first level of depression—no/minimal depression, which corresponded with the PHQ-9 scores ranging from 0 to 5, as the reference group. Trend analyses were conducted by entering the level of depression as a continuous variable and rerunning the corresponding regression models.

In all analyses, the complexity of the sampling design was taken into consideration by specifying primary sampling units (PSUs), strata, and weights using the R package “survey” (version 4.1-1). A good rule of thumb is to use “the least common denominator” where the variable of interest that was collected on the smallest number of respondents is the “least common denominator”. That is to say, we must use the weight of the smallest subpopulation that includes all the variables we want to include in our analysis. Reviewing the documentation file for each component included in our analysis, we used MEC exam weights to generate nationally representative estimates (27–29). To account for multiple testing, the method of Benjamini–Hochberg was used to correct the false discovery rate (FDR), and a two-sided FDR-adjusted *P*-value (i.e., q-value) <0.05, corresponding to an FDR of 5%, was deemed statistically significant for testing the study's hypotheses.

## Results

### Sample characteristics

A total of 34,079 NHANES participants aged 20–85 years old during the 2005–2018 period were interviewed. The 3,161 (9.28%) participants with missing data, and the final analysis unweighted sample consisted of 30,918 participants, representing 189.00 million non-institutionalized residents of the United States. Of those, 2,700 people (weighted proportion of 7.59%) reported symptoms of depression from moderate to severe, 1,231 people (weighted proportion of 3.39%) reported CHD, and 1,149 people (weighted proportion of 2.80%) reported stroke. These translated to 14.34 million, 6.41 million, and 5.28 million adults in the general population, respectively.

The sociodemographic and clinical characteristics among the weighted population are shown in Table 1. Of note, depression was especially prevalent among those who were Black, female participants, in the 41–60 age bracket, from disadvantaged socio-economic backgrounds (marital status other than married, lower levels of education, lower family income, etc.), current smokers, extremely obese, and those who were more likely to be comorbid with CHD, stroke, hypertension, CHF, angina, heart attack, arthritis, CKD, COPD, diabetes, hyperlipidemia, and Parkinson's disease compared to the non-depression group.

**Table 1:** Baseline characteristics of study participants^a^.

**Characteristic**	**Total**	**No depression** **(PHQ-9 < 10)**	**Depression** **(PHQ-9 ≥10)**	**Missing data,** **number (%)**	**Raw *P* value**	**q value**
Gender (%)				0 (0.00)	< 0.0001	< 0.0001
Male	49.37 (47.20, 51.54)	50.29 (49.70, 50.89)	35.26 (32.72, 37.80)			
Female	50.63 (48.37, 52.89)	49.71 (49.11, 50.30)	64.74 (62.20, 67.28)			
Age (%)				0 (0.00)	< 0.0001	< 0.0001
[20, 30]	19.88 (18.74, 21.01)	19.95 (18.93, 20.96)	18.78 (16.18, 21.37)			
[31, 40]	17.55 (16.64, 18.47)	17.56 (16.84, 18.29)	17.43 (15.19, 19.67)			
[41, 50]	19.37 (18.15, 20.60)	19.23 (18.43, 20.03)	21.61 (19.29, 23.93)			
[51, 60]	18.76 (17.57, 19.95)	18.57 (17.78, 19.36)	21.68 (19.36, 24.00)			
[61, 70]	13.69 (12.75, 14.63)	13.73 (13.04, 14.42)	13.09 (11.16, 15.01)			
[71, 80]	10.34 (9.63, 11.05)	10.54 (9.95, 11.12)	7.30 (5.92, 8.68)			
[81, 85]	0.41 (0.30, 0.51)	0.43 (0.32, 0.54)	0.12 (0.00, 0.26)			
Ethnicity (%)				0 (0.00)	< 0.0001	< 0.0001
White	68.72 (63.66, 73.78)	69.05 (66.61, 71.49)	63.74 (60.00, 67.49)			
Black	10.43 (9.37, 11.49)	10.23 (8.96, 11.51)	13.39 (11.47, 15.30)			
Mexican	8.35 (7.15, 9.55)	8.38 (7.08, 9.67)	7.92 (6.05, 9.80)			
Other	12.50 (11.55, 13.45)	12.34 (11.24, 13.44)	14.95 (12.75, 17.15)			
Marital status (%)				19 (0.06)	< 0.0001	< 0.0001
Married	55.85 (52.66, 59.05)	57.11 (55.81, 58.40)	36.65 (33.72, 39.59)			
Living with partner	8.20 (7.57, 8.82)	8.01 (7.47, 8.55)	11.06 (9.37, 12.75)			
Separated	2.37 (2.15, 2.59)	2.13 (1.91, 2.36)	5.96 (4.87, 7.05)			
Divorced	10.41 (9.75, 11.06)	9.90 (9.41, 10.40)	18.06 (16.25, 19.88)			
Widowed	5.52 (5.13, 5.91)	5.40 (5.08, 5.73)	7.34 (5.96, 8.71)			
Never married	17.66 (16.68, 18.63)	17.44 (16.35, 18.53)	20.93 (18.79, 23.07)			
Educational level (%)				26 (0.08)	< 0.0001	< 0.0001
College graduate or above	29.77 (27.44, 32.10)	30.85 (29.02, 32.69)	13.21 (10.83, 15.59)			
Some college or AA degree	31.79 (30.24, 33.35)	31.68 (30.66, 32.70)	33.47 (30.66, 36.28)			
High school graduate	23.27 (21.79, 24.75)	23.00 (22.03, 23.97)	27.42 (24.90, 29.95)			
9–11th grade	10.18 (9.32, 11.04)	9.72 (8.93, 10.50)	17.31 (15.28, 19.34)			
<9th Grade	4.98 (4.50, 5.47)	4.75 (4.27, 5.22)	8.59 (7.20, 9.97)			
Poverty income ratio (%)				0 (0.00)	< 0.0001	< 0.0001
High income	35.17 (32.57, 37.77)	36.35 (34.60, 38.11)	17.12 (14.45, 19.78)			
Middle income	27.15 (25.48, 28.82)	27.52 (26.41, 28.63)	21.50 (19.06, 23.93)			
Nearly poor	18.76 (17.65, 19.87)	18.22 (17.30, 19.14)	27.05 (24.38, 29.73)			
Poor	12.52 (11.69, 13.35)	11.53 (10.71, 12.35)	27.56 (24.89, 30.23)			
Unknown	6.40 (5.84, 6.97)	6.38 (5.84, 6.92)	6.77 (5.41, 8.14)			
Smoking status (%)				17 (0.05)	< 0.0001	< 0.0001
Never	54.65 (52.29, 57.01)	55.74 (54.62, 56.86)	37.95 (34.77, 41.13)			
Former	25.16 (23.54, 26.77)	25.38 (24.49, 26.26)	21.76 (19.22, 24.31)			
Current	20.20 (19.02, 21.37)	18.88 (18.04, 19.72)	40.29 (37.21, 43.36)			
Body mass index (%)				335 (0.98)	0.003	0.003
Normal	27.98 (26.50, 29.46)	28.28 (27.33, 29.24)	23.30 (20.54, 26.06)			
Overweight	70.54 (67.30, 73.78)	70.26 (69.24, 71.28)	74.77 (72.03, 77.51)			
Low	1.48 (1.30, 1.67)	1.46 (1.28, 1.63)	1.93 (0.91, 2.95)			
CHD (%)				0 (0.00)	< 0.001	0.005
No	96.61 (92.48, 100.74)	96.72 (96.40, 97.04)	94.91 (93.63, 96.18)			
Yes	3.39 (3.03, 3.75)	3.28 (2.96, 3.60)	5.09 (3.82, 6.37)			
Hypertension (%)				0 (0.00)	< 0.0001	< 0.0001
No	61.95 (59.08, 64.82)	62.62 (61.57, 63.67)	51.74 (49.22, 54.26)			
Yes	38.05 (36.15, 39.95)	37.38 (36.33, 38.43)	48.26 (45.74, 50.78)			
CHF (%)				55 (0.16)	< 0.0001	< 0.0001
No	97.81 (93.62, 102.00)	98.01 (97.81, 98.21)	94.69 (93.65, 95.72)			
Yes	2.19 (1.97, 2.42)	1.99 (1.79, 2.19)	5.31 (4.28, 6.35)			
Angina (%)				71 (0.21)	< 0.0001	< 0.0001
No	97.84 (93.66, 102.02)	98.02 (97.82, 98.23)	95.05 (93.86, 96.24)			
Yes	2.16 (1.93, 2.39)	1.98 (1.77, 2.18)	4.95 (3.76, 6.14)			
Heart attack (%)				35 (0.10)	< 0.0001	< 0.0001
No	96.71 (92.59, 100.84)	96.88 (96.61, 97.16)	94.09 (92.95, 95.23)			
Yes	3.29 (2.97, 3.60)	3.12 (2.84, 3.39)	5.91 (4.77, 7.05)			
Stroke (%)				36 (0.11)	< 0.0001	< 0.0001
No	97.20 (93.04, 101.37)	97.50 (97.28, 97.71)	92.76 (91.47, 94.04)			
Yes	2.80 (2.55, 3.04)	2.50 (2.29, 2.72)	7.24 (5.96, 8.53)			
Arthritis (%)				58 (0.17)	< 0.0001	< 0.0001
No	73.88 (70.69, 77.07)	75.02 (74.09, 75.95)	56.42 (53.80, 59.05)			
Yes	26.12 (24.57, 27.67)	24.98 (24.05, 25.91)	43.58 (40.95, 46.20)	1686 (4.95)		
CKD (%)					< 0.001	0.0008
No	85.93 (82.10, 89.77)	86.12 (85.50, 86.73)	83.14 (81.36, 84.93)			
Yes	14.07 (13.29, 14.84)	13.88 (13.27, 14.50)	16.86 (15.07, 18.64)			
CKD prognosis (%)				2001 (5.87)	< 0.0001	0.0005
Low risk	85.93 (82.10, 89.77)	86.12 (85.50, 86.73)	83.14 (81.36, 84.93)			
Moderate risk	10.21 (9.59, 10.83)	10.14 (9.64, 10.63)	11.31 (9.72, 12.90)			
High risk	2.54 (2.32, 2.77)	2.49 (2.27, 2.70)	3.40 (2.65, 4.15)			
Very high risk	1.31 (1.20, 1.43)	1.26 (1.14, 1.38)	2.15 (1.60, 2.71)			
Diabetes (%)				633 (1.86)	< 0.0001	< 0.0001
No	81.46 (77.84, 85.08)	81.87 (81.14, 82.59)	75.32 (73.35, 77.28)			
Diabetes	13.38 (12.62, 14.13)	12.97 (12.37, 13.58)	19.57 (17.75, 21.40)			
IGT	5.16 (4.74, 5.58)	5.16 (4.81, 5.51)	5.11 (4.04, 6.18)			
Hyperlipidemia (%)				1 (0.00)	0.0001	0.0001
No	29.30 (27.96, 30.63)	29.68 (28.71, 30.64)	23.51 (20.49, 26.53)			
Yes	70.70 (67.29, 74.12)	70.32 (69.36, 71.29)	76.49 (73.47, 79.51)			
Parkinson (%)				18 (0.05)	< 0.0001	0.0001
No	99.03 (94.81, 103.25)	99.17 (99.02, 99.32)	96.91 (95.87, 97.95)			
Yes	0.97 (0.80, 1.14)	0.83 (0.68, 0.98)	3.09 (2.05, 4.13)			

Data are expressed as weighted proportions (95% CI) for categorical variables and as weighted means (95% CI) for continuous variables.

CHD, coronary heart disease; CHF, congestive heart failure; CKD, chronic kidney disease; COPD, chronic obstructive pulmonary disease; IGT, impaired glucose tolerance; PHQ-9, the nine-item Patient Health Questionnaire.

^a^Two-sided *P*-values show result of univariate comparisons between depressed participants and participants who were not depressed. All categorical variables were tested with the χ^2^-test.

### Subgroup analyses

**Figure 1:**
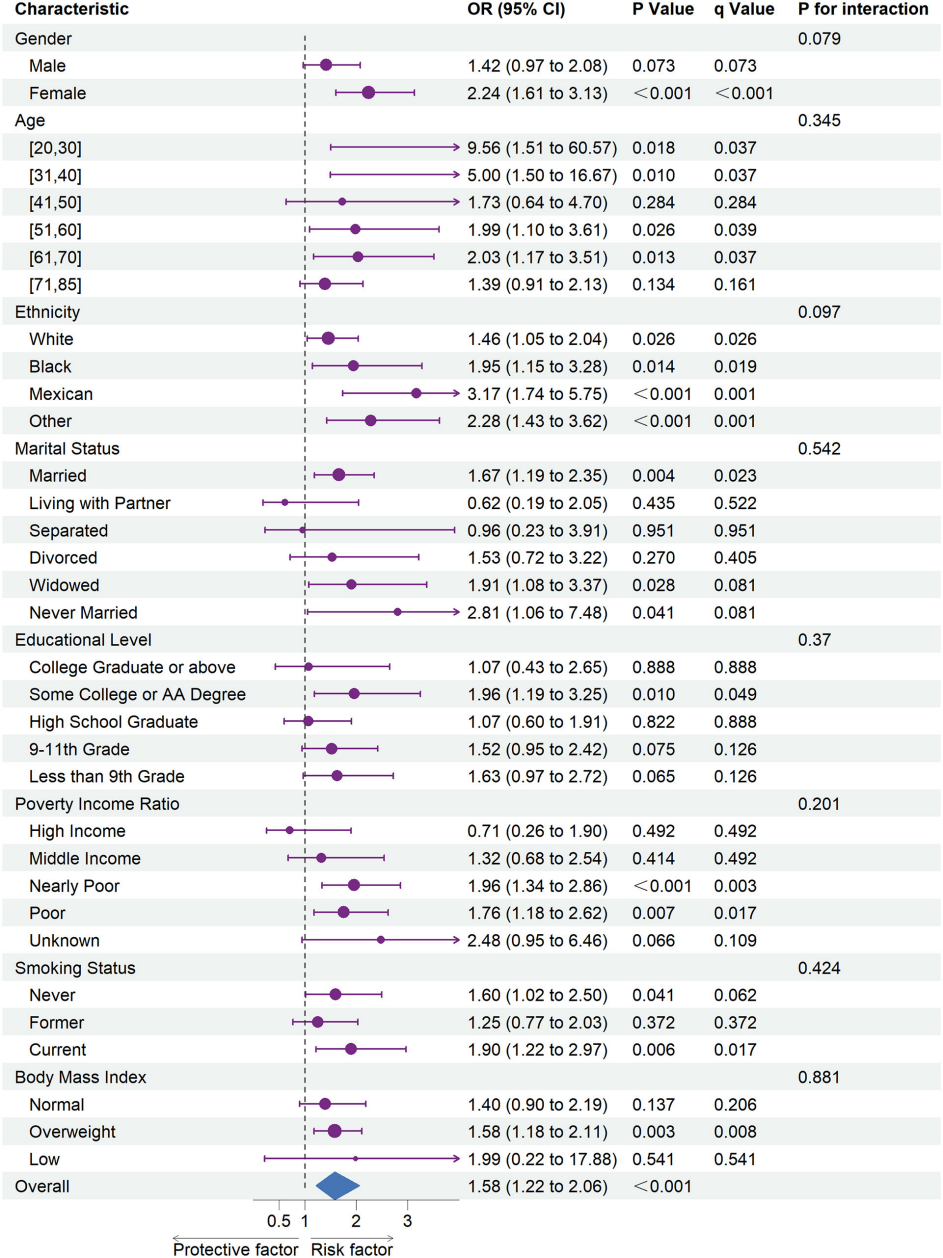
The association between depression and coronary heart disease risk in each subgroups. OR, odds ratio; CI, confidence interval.

**Figure 2:**
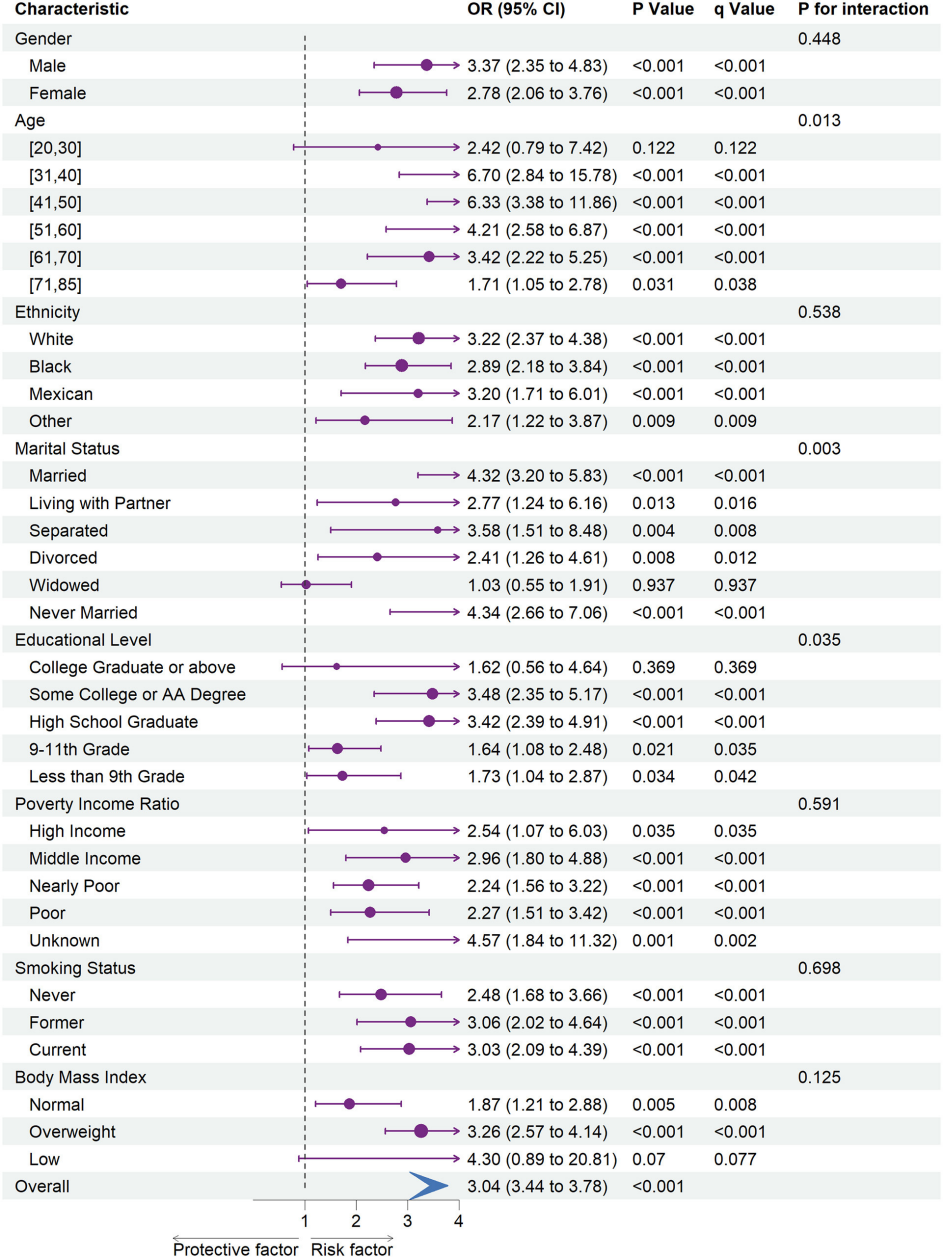
The association between depression and stroke risk in each subgroups. OR, odds ratio; CI, confidence interval.

Figures 1, 2 shows the results of subgroup analysis using univariable weighted logistic regression analyses.

Subgroup analysis revealed an increased risk of CHD (OR = 1.58; 95% CI = 1.22 to 2.06) and stroke (OR = 3.04; 95% CI = 3.44 to 3.78) associated with depression in overall participants. Specifically, an increased risk of CHD was associated with depression in female participants, every ethnic group, and among participants of the age ranges 20–30, 31–40, 51–60, or 61–70. Besides, we also observed an increased risk of CHD associated with depression in participants whose marital status was married, widowed, or never married, whose education level was some college or AA degree, whose family PIR was nearly poor or poor, whose smoking status was never or current, and whose BMI was categorized as overweight.

Moreover, an increased risk of stroke was associated with depression within each gender, ethnicity, family PIR, smoking status, and among participants aged range from 31 to 85. Besides, we found that stroke risk was not associated with depression, among participants whose education level was college graduate or above, whose marital status was widowed, or whose BMI was categorized as low.

In subgroup analyses (Figures 1, 2), statistically significant interactions were not observed between depression and any study covariates in relation to CHD (all P for interaction > 0.05), but statistically significant interactions were noted between depression and age, marital status, and education level in relation to stroke (P for interaction = 0.013, 0.003, 0.035, respectively) despite the lack of interaction for other variables.

### The association of depression level with CHD, stroke risk

Results of weighted logistic regression analyses of depression severity in relation to the risk of CHD and stroke are shown in Table 2. There was a statistically significant association between depression severity and increased risk of CHD and stroke in models 1, 2, and 3, and those linear trend tests were all statistically significant (all *P* for trend < 0.001). For example, the result in model 3 showed that the risk of having CHD increased by 38, 120, 84, and 275% for mild, moderate, moderately severe, or severe depression, respectively, compared to no/minimal depression. Similarly, weighted logistic regression model 3 indicated that each additional level increase in the severity of depression raised the risks of stroke by 79, 137, 236, and 437%, respectively after adjustment for covariates.

**Table 2:** Crude and adjusted association between depression level and increased coronary heart disease and stroke risk.

**Model**	**Severity of depression**					***P*** **value for trend**
	**No/minimal** **prevalence = 77.24** **(73.71,80.77)**	**Mild** **prevalence = 15.17** **(14.42, 15.92)**	**Moderate** **prevalence = 4.83** **(4.44, 5.22)**	**Moderately severe** **prevalence = 1.98** **(1.76, 2.20)**	**Severe** **prevalence = 0.78** **(0.67, 0.88)**	
**Coronary heart disease**							
Model 1 (or)	1.00 (Reference)	1.19 (0.92 to 1.53)	1.76 (1.27 to 2.46)	1.47 (0.95 to 2.27)	2.55 (1.54 to 4.23)	<0.001
Raw *p* values		0.181	0.001	0.086	<0.001	
q value		0.181	0.002	0.114	0.002	
Model 2 (or)	1.00 (Reference)	1.50 (1.16 to 1.94)	2.55 (1.77 to 3.68)	2.09 (1.36 to 3.22)	4.58 (2.50 to 8.37)	<0.001
Raw p value		0.003	<0.001	0.001	<0.001	
q value		0.003	<0.001	0.002	<0.001	
Model 3 (or)	1.00 (Reference)	1.38 (1.06 to 1.79)	2.20 (1.53 to 3.16)	1.84 (1.20 to 2.81)	3.75 (2.07 to 6.78)	<0.001
Raw *p* values		0.019	<0.001	0.007	<0.001	
q value		0.019	<0.001	0.009	<0.001	
**Stroke**							
Model 1 (or)	1.00 (Reference)	1.95 (1.61 to 2.35)	2.72 (2.01 to 3.69)	3.77 (2.67 to 5.32)	5.94 (3.91 to 9.02)	<0.001
Raw *p* values		<0.001	<0.001	<0.001	<0.001	
q value		<0.001	<0.001	<0.001	<0.001	
Model 2 (or)	1.00 (Reference)	2.09 (1.72 to 2.53)	3.16 (2.28 to 4.39)	4.62 (3.18 to 6.69)	7.86 (5.08 to 12.16)	<0.001
Raw *p* value		<0.001	<0.001	<0.001	<0.001	
q value		<0.001	<0.001	<0.001	<0.001	
Model 3 (or)	1.00 (Reference)	1.79 (1.47 to 2.18)	2.37 (1.69 to 3.32)	3.36 (2.26 to 4.98)	5.37 (3.42 to 8.44)	<0.001
Raw *p* values		<0.001	<0.001	<0.001	<0.001	
q value		<0.001	<0.001	<0.001	<0.001	

Data are expressed as weighted means (95% CI) for continuous variables.

Model 1: Unadjusted model.

Model 2: Adjusted for age, gender, and ethnicity.

Model 3: Adjusted for age, gender, ethnicity, marital status, family PIR, education, smoking status, and BMI.

OR, odds ratio; PIR, poverty income ratio; BMI, body mass index.

### Cause-specific mortality analyses

The leading causes of death in different depression severity groups are shown in Table 3. Among them, cardiovascular mortality rates were 1.91, 2.46, 2.78, 2.94, and 1.05% for no/minimal, mild, moderate, moderately severe, or severe depression, respectively. Moreover, the prevalence of all-cause mortality was 6.62, 8.60, 9.48, 10.77, and 10.42% for no/minimal, mild, moderate, moderately severe, and severe depression, respectively.

**Table 3:** The weighted percentages of leading causes of death in different depression severity groups.

**Cause of death**	**No/Minimal**	**Mild**	**Moderate**	**Moderately severe**	**Severe**
Diseases of heart (%)	534 (1.57)	145 (2.03)	54 (2.47)	21 (2.64)	5 (1.05)
Cerebrovascular diseases (%)	124 (0.34)	27 (0.43)	8 (0.31)	3 (0.30)	0 (0.00)
Influenza and pneumonia (%)	42 (0.12)	5 (0.06)	2 (0.11)	3 (0.27)	0 (0.00)
Chronic lower respiratory diseases (%)	106 (0.33)	40 (0.75)	11 (0.45)	9 (1.13)	2 (0.61)
Nephritis, nephrotic syndrome and nephrosis (%)	34 (0.08)	10 (0.14)	7 (0.25)	4 (0.51)	0 (0.00)
Diabetes mellitus (%)	68 (0.19)	21 (0.39)	9 (0.63)	3 (0.19)	2 (0.69)
Malignant neoplasms (%)	555 (1.77)	119 (1.88)	43 (1.73)	15 (1.51)	7 (1.25)
Alzheimer's disease (%)	64 (0.19)	12 (0.15)	1 (0.02)	0 (0.00)	0 (0.00)
Accidents (unintentional injuries) (%)	62 (0.24)	15 (0.34)	4 (0.16)	2 (0.45)	6 (1.79)
All other causes (residual) (%)	604 (1.79)	139 (2.43)	64 (3.35)	25 (3.77)	11 (5.03)
All-cause (%)	2,193 (6.62)	533 (8.60)	203 (9.48)	85 (10.77)	33 (10.42)

Data are expressed as sample sizes (weighted percentage) for continuous variables.

The unweighted sample consisted of 30918 participants and represented 189.00 million non-institutionalized residents of the United States.

### Survival analysis

All the participants were followed up after the home interview. After excluding the participants with CVDs, cancer, or malignancy at baseline in the survival analyses, the median follow-up time in the population-based cohort was 92.00 months (interquartile range: 52–132 months).

The weighted Cox regression analysis results are shown in Tables 4, 5, performed to estimate the relationships between the severity of depression and their risk of having all-cause and cardiovascular mortality, respectively.

**Table 4:** Crude and adjusted association between depression level and all-cause mortality.

**Model**	**Severity of depression**					***P*** **value for trend**
	**No/Minimal**	**Mild**	**Moderate**	**Moderately severe**	**Severe**	
Model 1 (HR)	1.00 (Reference)	1.36 (1.11 to 1.67)	1.55 (1.20 to 2.01)	1.99 (1.34 to 2.94)	2.08 (1.08 to 4.00)	<0.001
*P* value		0.003	0.001	0.001	0.028	
q value		0.004	0.002	0.002	0.028	
Model 2 (HR)	1.00 (Reference)	1.63 (1.31 to 2.01)	1.99 (1.54 to 2.56)	2.67 (1.73 to 4.11)	3.25 (1.71 to 6.20)	<0.001
*P* values		<0.001	<0.001	<0.001	<0.001	
q value		<0.001	<0.001	<0.001	<0.001	
Model 3 (HR)	1.00 (Reference)	1.38 (1.11 to 1.72)	1.41 (1.09 to 1.82)	1.95 (1.30 to 2.93)	2.08 (1.03 to 4.23)	<0.001
*P* values		0.003	0.010	0.001	0.042	
q value		0.003	0.010	0.001	0.042	

Data are expressed as weighted means (95% CI) for continuous variables.

Model 1: Unadjusted model.

Model 2: Adjusted for age, gender, and ethnicity.

Model 3: Adjusted for age, gender, ethnicity, marital status, family PIR, education, smoking status, and BMI.

HR, hazard ratio; PIR, poverty income ratio; BMI, body mass index.

**Table 5:** Crude and adjusted association between depression level and cardiovascular mortality.

**Model**	**Severity of depression**				
	**No/minimal**	**Mild**	**Moderate**	**Moderately severe**	**Severe**
Model 1 (HR)	1.00 (Reference)	1.47 (1.18 to 1.83)	1.74 (1.20 to 2.52)	2.07 (1.22 to 3.50)	0.85 (0.30 to 2.43)
*P* value		0.001	0.004	0.007	0.758
q value		0.002	0.007	0.009	0.758
Model 2 (HR)	1.00 (Reference)	1.75 (1.38 to 2.22)	2.27 (1.57 to 3.30)	3.06 (1.81 to 5.16)	1.36 (0.45 to 4.09)
*P* value		<0.001	<0.001	<0.001	0.586
q value		<0.001	<0.001	<0.001	0.586
Model 3 (HR)	1.00 (Reference)	1.49 (1.16 to 1.91)	1.68 (1.15 to 2.45)	2.37 (1.39 to 4.06)	0.89 (0.29 to 2.76)
*P* value		0.002	0.007	0.002	0.845
q value		0.003	0.010	0.003	0.845

Data are expressed as weighted means (95% CI) for continuous variables.

Model 1: Unadjusted model.

Model 2: Adjusted for age, gender, and ethnicity.

Model 3: Adjusted for age, gender, ethnicity, marital status, family PIR, education, smoking status, and BMI.

HR, hazard ratio; PIR, poverty income ratio; BMI, body mass index.

The results from a series of weighted Cox regression analyses in Table 4 consistently indicated that participants with a more severe degree of depression were at a higher risk of all-cause death. For instance, weighted multivariable Cox proportional hazard model 3 indicated that each additional level increase in the severity of depression raised the risks of all-cause death by 38, 41, 95, and 108%, respectively, after adjustment for covariates. Trend analyses were all statistically significant (all P for trend < 0.001).

However, a series of weighted Cox regression analysis results in Table 5 consistently suggests that with the exception of severe depression, the hazard of cardiovascular mortality increased with each additional level increase of depression. Using weighted multivariable Cox proportional hazard model 3 as an example, compared to no/minimal depression, the risks of cardiovascular death were increased by 49, 68, and 137% for mild, moderate, and moderately severe depression, respectively, after multivariable adjustment.

**Figure 3:**
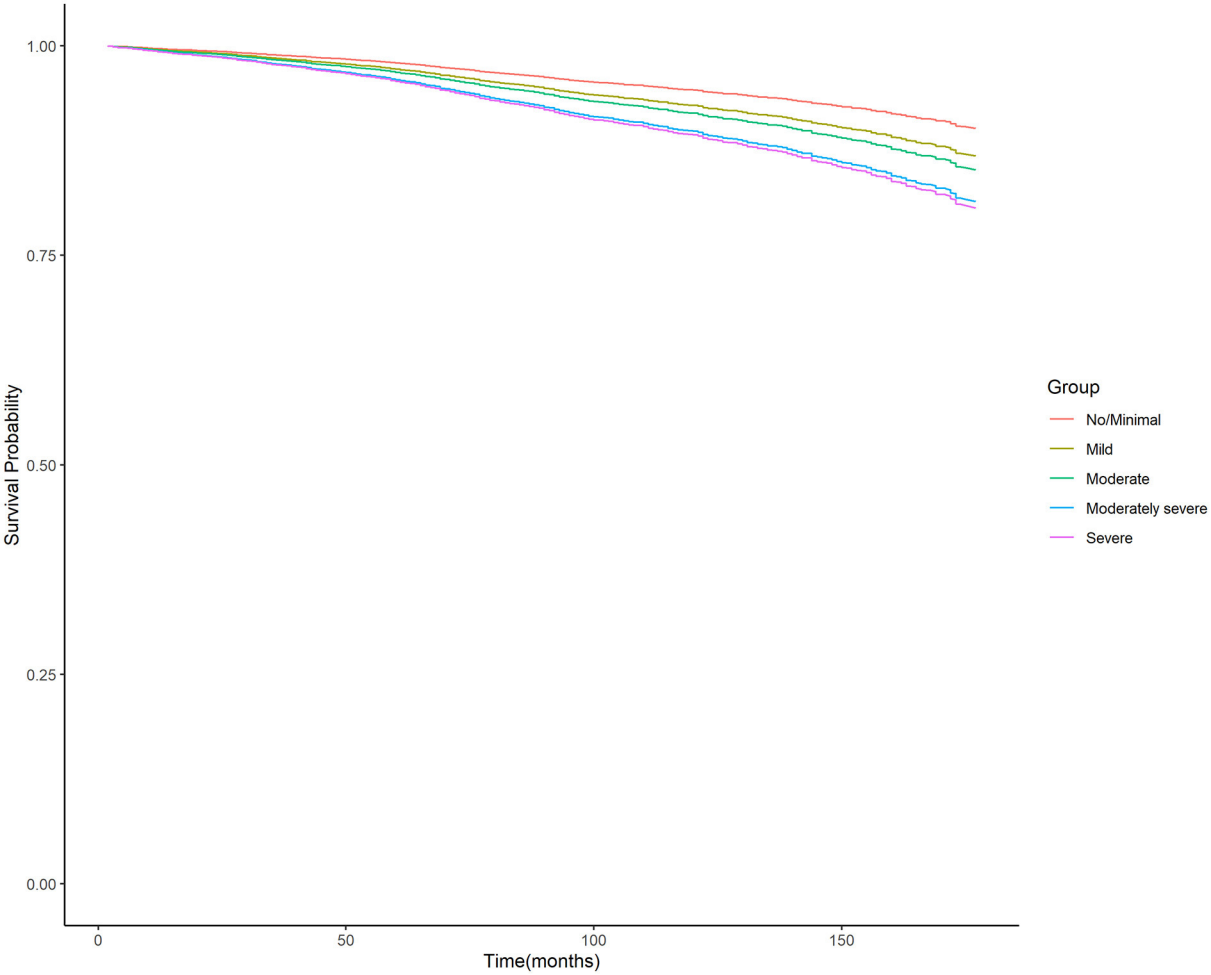
Unadjusted survival curves of weighted cox proportional hazard model for all-cause mortality. PHQ-9, nine-item Patient Health Questionnaire.

**Figure 4:**
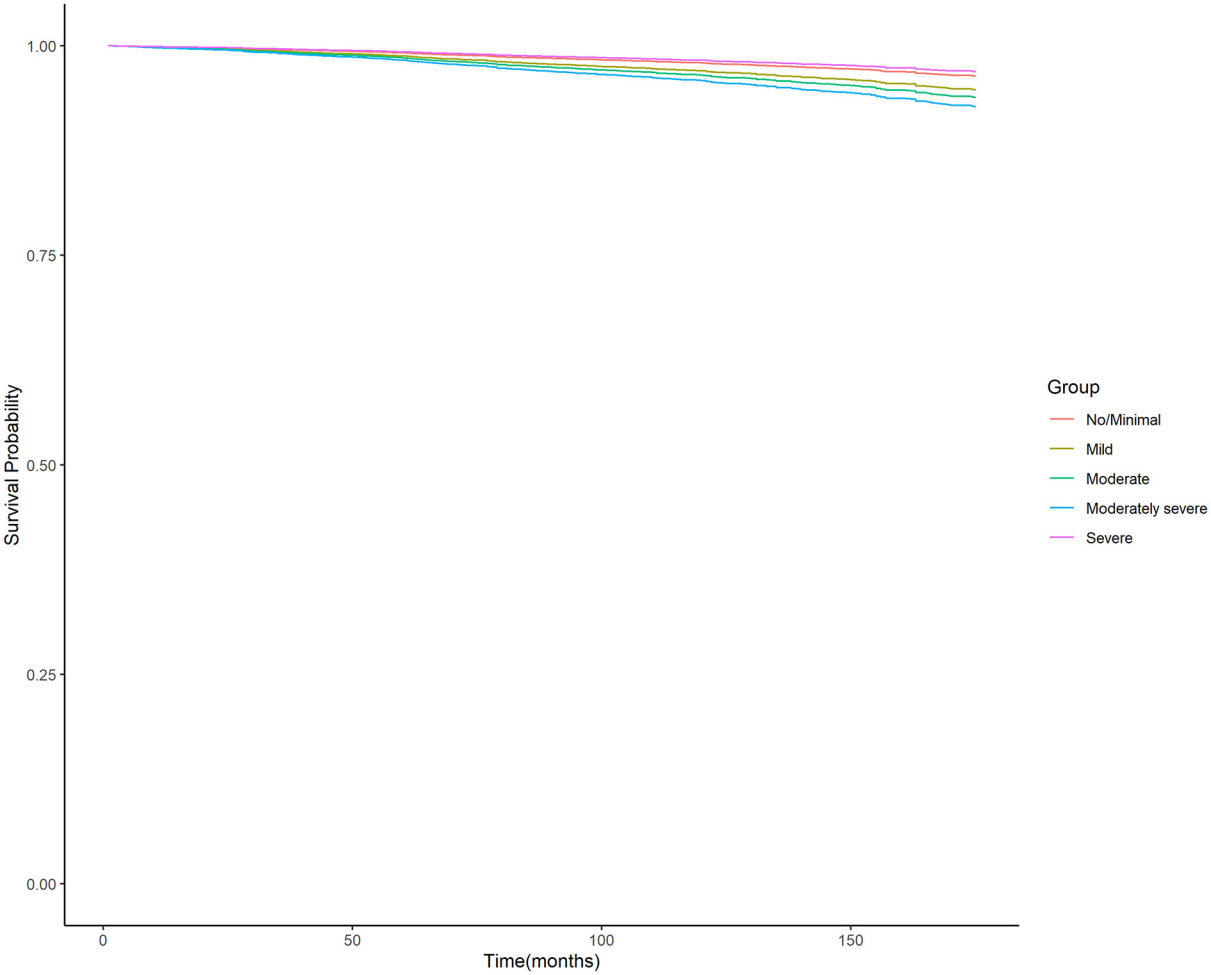
Unadjusted survival curves of weighted cox proportional hazard model for cardiovascular mortality. PHQ-9, nine-item Patient Health Questionnaire.

Moreover, the unadjusted survival curves of weighted Cox proportional hazards models for all-cause and cardiovascular mortality (Figures 3, 4) were consistent with the aforementioned findings.

## Discussion

In our study, performed on a nationally representative cohort of the United States population, we found that the level of depression was independently associated with higher risk of having CHD, stroke, and all-cause and cardiovascular mortality.

Cardiovascular diseases are often comorbid with depression. Cohen et al. reported that 20% of patients with CVDs had moderate to severe depression, and another 20% of patients with CVDs had mild to moderate depression, which is approximately two to three times the rate of the general population (30). In addition, a meta-analysis with a median follow-up of 8.4 years revealed that the cumulative incidence of CVDs in patients with severe mental illness (SMI) was 3.6% (95% CI = 2.7% to 5.3%), which was significantly higher than in people without SMI (HR = 1.78; 95% CI = 1.60 to 1.80); compared to the control group, patients with depression had 1.72 times the risk of CVDs after controlling for confounding variables (31).

The mechanisms underlying the common dual comorbidities of depression and CVDs were complex, and current research suggested that depression and CVDs may be linked by biological and behavioral mechanisms, including *via* metabolic syndrome—which is prevalent in depression, type 2 diabetes, increased visceral adipose thickness, changes in cortisol levels due to the dysregulation of the hypothalamic–pituitary–adrenal (HPA) axis and unhealthy lifestyle habits (smoking, poor diet, lack of exercise, etc.) (32–37). Moreover, in recent years, studies also reported that cardiovascular traits such as blood pressure and arterial stiffness were influenced by and associated with depression (38, 39). Of note is a recent study by Mutz et al., which reported that depression resulted in a 1 mmHg lower systolic blood pressure (SBP) at age 45 and a 2.5 mmHg lower SBP at age 65 in male participants (38). However, it was also worth noting that the mean values of SBP in male participants were all near 140 mmHg, whether in healthy control or depression case in that study. In this context, the effect of such a small SBP difference on the coronary arteries was negligible in male participants. Thus, the present study is not in contradiction with the Mutz et al. study. In addition, studies have shown that these two diseases may be driven by a common genetic susceptibility, and each disease increased the risk of the other (40).

Previous studies had demonstrated that depression was identified as a risk factor for CVDs (41), while the association between specific severity of depression and CHD, stroke risk as well as mortality is still unknown. For this reason, further research was undertaken in this study, and we found out that with each additional level increase of depression, the implications of such an increase in the risk of having CHD, stroke, and mortality were vast.

Therefore, by understanding the relationship and degree of impact, we can properly identify, prevent, and treat CVDs, and we will be able to create policies and strategies to help decrease CVDs and improve lives by tackling mental health. This emphasizes the importance of regular screening for cardiovascular risk factors in patients with depression.

Interestingly, we also observed that participants with higher depression levels had a higher risk of all-cause mortality; a previous study showed that the mortality for depression patients with concomitant CVD whose depression was well-treated, treatment-resistance, and under-treated were 2.4, 5, and 6.9%, respectively (42, 43). These findings suggested that enhanced depression treatment reduces the risk of death.

In analyses of the association between depression level and cardiovascular death, we found that the hazard of cardiovascular death was the lowest in the severe depression group. This phenomenon may be explained by the result of the death cause analysis in our study. We found that the leading cause in this group was all other causes (residual) (5.03%) followed by accidents (unintentional injuries) (1.79%), malignant neoplasms (1.25%), and heart diseases (1.05%).

In summary, patients with depression may need early and/or more primary prevention efforts for CVDs to reduce their excessive CVDs burden. For clinicians, this means a need for effective collaboration with primary care clinicians and cardiologists. Early treatment is more likely to modify the risk factor in the progression of cardiac disease than to reverse these risk factors after the first heart attack (36).

Systematic monitoring of depression was particularly beneficial in cases where physical illness overlaps with depressive symptoms (44). Serial assessment of depressive symptoms with the PHQ-9 or similar methods can improve the efficiency of antidepressant treatment. It also documents the relationship between depressive symptoms and specific physiological indicators of CVDs (45).

It follows then that the first limitation in our study is that PHQ-9 was measured only once and was not followed up for subsequent changes in depression level, cumulative depression burden, incident depression, or time-varying associations with outcomes.

Second, some measures in this study, including the diagnosis of CHD, stroke, and any symptoms of depression in PHQ-9, were self-reported by the participants, which may have introduced recall bias to the associations.

Third, the NHANES program suspended field operations in March 2020 due to the coronavirus disease 2019 (COVID-19) pandemic. As a result, data collection for the NHANES 2019–2020 cycle was not completed and the collected data are not nationally representative, and the PHQ-9 information had not been included in the home interview since 2005, As a result, we only included participants from 2005 to 2018.

Fourth, the application of the competitive risk model in the survival analyses cannot be performed due to the complex, stratified multistage, probability cluster design of the NHANES survey.

Fifth, some of the covariates included as covariates in the multivariable-adjusted model (e.g., BMI) were likely on the causal pathway linking depression to cardiovascular outcomes. Therefore, interpretation of the present findings needs to also consider the risk of potential over adjustment bias.

## Conclusion

In conclusion, our study confirmed that the level of depression was strongly associated with CHD, stroke, and all-cause and cardiovascular mortality, even after accounting for other factors that could impact risk, including variables of age, gender, ethnicity, income, education, BMI, marital, and smoking status.

## Data availability statement

Publicly available datasets were analyzed in this study. This data can be found here: https://www.cdc.gov/nchs/nhanes/index.htm and https://www.cdc.gov/nchs/data-linkage/mortality-public.htm.

## Ethics statement

The studies involving human participants were reviewed and approved by the NCHS Research Ethics Review Board. The patients/participants provided their written informed consent to participate in this study.

## Author contributions

RS had full access to all of the data in the study and takes responsibility for the integrity of the data and the accuracy of the data analysis. Concept and design: RS, NZ, and JW. Drafting of the manuscript: RS and NZ. Critical revision of the manuscript for important intellectual content: RS, NZ, TZ, JW, PG, and SS. Statistical analysis: RS and SS. Obtained funding and supervision: TZ. Administrative, technical, or material support: TZ, DL, and PG. Acquisition, analysis, or interpretation of data: all authors. All authors contributed to the article and approved the submitted version.

## Funding

This work was supported by the Beijing Municipal Science and Technology Commission Program (Nos. Z171100001017203 and D181100000218005).

## Conflict of interest

The authors declare that the research was conducted in the absence of any commercial or financial relationships that could be construed as a potential conflict of interest.

## Publisher's note

All claims expressed in this article are solely those of the authors and do not necessarily represent those of their affiliated organizations, or those of the publisher, the editors and the reviewers. Any product that may be evaluated in this article, or claim that may be made by its manufacturer, is not guaranteed or endorsed by the publisher.

## References

[1] World Health Organization. Depression and Other Common Mental Disorders: Global Health Estimates. (2017). Available online at: https://apps.who.int/iris/bitstream/handle/10665/254610/WHO-MSD-MER-2017.2-eng.pdf (accessed May 20, 2022).

[2] Loomba RS Aggarwal S Arora R. Depressive symptom frequency and prevalence of cardiovascular diseases-analysis of patients in the national health and nutrition examination survey. Am J Ther. (2015) 22:382–7. 10.1097/MJT.0000000000000043 25658955

[3] Liu N Pan XF Yu C Lv J Guo Y Bian Z. Association of major depression with risk of ischemic heart disease in a mega-cohort of chinese adults: the china kadoorie biobank study. J Am Heart Assoc. (2016) 5:e004687. 10.1161/JAHA.116.004687 28003250 PMC5210415

[4] Jiang W Krishnan RR O'Connor CM. Depression and heart disease: evidence of a link, and its therapeutic implications. CNS Drugs. (2002) 16:111–27. 10.2165/00023210-200216020-00004 11825102

[5] World Health Organization. The global burden of disease: 2004 update. (2008). Available online at: https://apps.who.int/iris/bitstream/handle/10665/43942/9789241563710_eng.pdf (accessed May 20, 2022).

[6] Sanchis-Gomar F Perez-Quilis C Leischik R Lucia A. Epidemiology of coronary heart disease and acute coronary syndrome. Ann Transl Med. (2016) 4:256. 10.21037/atm.2016.06.33 27500157 PMC4958723

[7] Ditmars HL Logue MW Toomey R McKenzie RE Franz CE Panizzon MS. Associations between depression and cardiometabolic health: A 27-year longitudinal study. Psychol Med. (2021) 12:1–11. 10.1017/S003329172000505X 33431106 PMC8547283

[8] Rajan S McKee M Rangarajan S Bangdiwala S Rosengren A Gupta R. Prospective urban rural epidemiology (PURE) study investigators. association of symptoms of depression with cardiovascular disease and mortality in low-, middle-, and high-income countries. JAMA Psychiatry. (2020) 77:1052–63. 10.1001/jamapsychiatry.2020.1351 32520341 PMC7287938

[9] Carney RM Freedland KE. Depression and coronary heart disease. Nat Rev Cardiol. (2017) 14:145–55. 10.1038/nrcardio.2016.181 27853162

[10] Pan A Sun Q Okereke OI Rexrode KM Hu FB. Depression and risk of stroke morbidity and mortality: a meta-analysis and systematic review. JAMA. (2011) 306:1241–9. 10.1001/jama.2011.1282 21934057 PMC3242806

[11] Li M Zhang XW Hou WS Tang ZY. Impact of depression on incident stroke: a meta-analysis. Int J Cardiol. (2015) 180:103–10. 10.1016/j.ijcard.2014.11.198 25438228

[12] Lichtman JH Froelicher ES Blumenthal JA Carney RM Doering LV Frasure-Smith N. Depression as a risk factor for poor prognosis among patients with acute coronary syndrome: systematic review and recommendations: a scientific statement from the American Heart Association. Circulation. (2014) 129:1350–69. 10.1161/CIR.0000000000000019 24566200

[13] Joynt KE Whellan DJ O'Connor CM. Depression and cardiovascular disease: mechanisms of interaction. Biol Psychiatry. (2003) 54:248–61. 10.1016/s0006-3223(03)00568-7 12893101

[14] Gan Y Gong Y Tong X Sun H Cong Y Dong X. Depression and the risk of coronary heart disease: a meta-analysis of prospective cohort studies. BMC Psychiatry. (2014) 14:371. 10.1186/s12888-014-0371-z 25540022 PMC4336481

[15] Seldenrijk A Vogelzangs N Batelaan NM Wieman I van Schaik DJ Penninx BJ. Depression, anxiety and 6-year risk of cardiovascular disease. J Psychosom Res. (2015) 78:123–9. 10.1016/j.jpsychores.2014.10.007 25454680

[16] National Center for Health Statistics. About the National Health and Nutrition Examination Survey. (2020). Available online at: https://www.cdc.gov/nchs/about/index.htm (accessed May 20, 2022).

[17] National Center for Health Statistics. Office of Analysis and Epidemiology. The Linkage of National Center for Health Statistics Survey Data to the National Death Index - 2019 Linked Mortality File (LMF): Methodology Overview and Analytic Considerations, March 2019. Hyattsville, Maryland. Available online at: https://www.cdc.gov/nchs/data/datalinkage/public-use-linked-mortality-file-description.pdf (accessed May 20, 2022).

[18] Centers for Disease Control and Prevention (CDC). National Center for Health Statistics (NCHS). National Health and Nutrition Examination Survey Methods and Analytic Guidelines. Hyattsville, MD: U.S. Department of Health and Human Services, Centers for Disease Control and Prevention. (2020). Available online at: https://www.cdc.gov/nchs/ahcd/survey_methods.htm (accessed May 20, 2022).

[19] Kroenke K Spitzer RL Williams JB. The PHQ-9: validity of a brief depression severity measure. J Gen Intern Med. (2001) 16:606–13. 10.1046/j.1525-1497.2001.016009606.x 11556941 PMC1495268

[20] Liu Y Ozodiegwu ID Yu Y Hess R Bie R. An association of health behaviors with depression and metabolic risks: Data from 2007 to 2014 U.S. National Health and Nutrition Examination Survey. J Affect Disord. (2017) 217:190–6. 10.1016/j.jad.2017.04.009 28412644

[21] Centers of Disease Control and prevention. Defining Adult Overweight and Obesity. Overweight & Obesity. (2021). Available online at: https://www.cdc.gov/obesity/basics/adult-defining.html (accessed May 20, 2022).

[22] Smolderen KG Strait KM Dreyer RP D'Onofrio G Zhou S Lichtman JH. Depressive symptoms in younger women and men with acute myocardial infarction: insights from the VIRGO study. J Am Heart Assoc. (2015) 4:e001424. 10.1161/JAHA.114.001424 25836055 PMC4579927

[23] Hagen EH Rosenström T. Explaining the sex difference in depression with a unified bargaining model of anger and depression. Evol Med Public Health. (2016) 2016:117–32. 10.1093/emph/eow006 26884416 PMC4804352

[24] Lorant V Deliège D Eaton W Robert A Philippot P Ansseau M. Socioeconomic inequalities in depression: a meta-analysis. Am J Epidemiol. (2003) 157:98–112. 10.1093/aje/kwf182 12522017

[25] Surtees PG Wainwright NW Luben RN Wareham NJ Bingham SA Khaw KT. Depression and ischemic heart disease mortality: evidence from the EPIC-Norfolk United Kingdom prospective cohort study. Am J Psychiatry. (2008) 165:515–23. 10.1176/appi.ajp.2007.07061018 18245176

[26] Meijer A Conradi HJ Bos EH Anselmino M Carney RM Denollet J. Adjusted prognostic association of depression following myocardial infarction with mortality and cardiovascular events: individual patient data meta-analysis. Br J Psychiatry. (2013) 203:90–102. 10.1192/bjp.bp.112.111195 23908341

[27] Johnson CL Paulose-Ram R Ogden CL Carroll MD Kruszon-Moran D Dohrmann SM. National health and nutrition examination survey: analytic guidelines, 1999-2010. Vital Health Stat 2. (2013) 161:1–24. 25090154

[28] Vallance JK Winkler EA Gardiner PA Healy GN Lynch BM Owen N. Associations of objectively-assessed physical activity and sedentary time with depression: NHANES (2005-2006). Prev Med. (2011) 53:284–8. 10.1016/j.ypmed.2011.07.013 21820466

[29] Loprinzi PD Cardinal BJ. Association between objectively-measured physical activity and sleep, NHANES 2005–2006. Ment Health Phys Act. (2011) 4:65–9. 10.1016/j.mhpa.2011.08.001

[30] Cohen BE Edmondson D Kronish IM. State of the art review: depression, stress, anxiety, and cardiovascular disease. Am J Hypertens. (2015) 28:1295–302. 10.1093/ajh/hpv047 25911639 PMC4612342

[31] Correll CU Solmi M Veronese N Bortolato B Rosson S Santonastaso P. Prevalence, incidence and mortality from cardiovascular disease in patients with pooled and specific severe mental illness: a large-scale meta-analysis of 3,211,768 patients and 113,383,368 controls. World Psychiatry. (2017) 16:163–80. 10.1002/wps.20420 28498599 PMC5428179

[32] van Agtmaal MJM Houben AJHM Pouwer F Stehouwer CDA Schram MT. Association of microvascular dysfunction with late-life depression: a systematic review and meta-analysis. JAMA Psychiatry. (2017) 74:729–39. 10.1001/jamapsychiatry.2017.0984 28564681 PMC5710252

[33] Pan A Lucas M Sun Q van Dam RM Franco OH Manson JE. Bidirectional association between depression and type 2 diabetes mellitus in women. Arch Intern Med. (2010) 170:1884–91. 10.1001/archinternmed.2010.356 21098346 PMC3065781

[34] Lee JI Busler JN Millett CE Principe JL Levin LL Corrigan A. Association between visceral adipose tissue and major depressive disorder across the lifespan: A scoping review [published online ahead of print, 2021 Sep 22]. Bipolar Disord. (2021). 10.1111/bdi.13130 34551182

[35] Francis J Chu Y Johnson AK Weiss RM Felder RB. Acute myocardial infarction induces hypothalamic cytokine synthesis. Am J Physiol Heart Circ Physiol. (2004) 286:H2264–71. 10.1152/ajpheart.01072.2003 15148057

[36] Vaccarino V Badimon L Bremner JD Cenko E Cubedo J Dorobantu M. Depression and coronary heart disease: 2018 position paper of the ESC working group on coronary pathophysiology and microcirculation. Eur Heart J. (2020) 41:1687–96. 10.1093/eurheartj/ehy913 30698764 PMC10941327

[37] Miller AH Raison CL. The role of inflammation in depression: from evolutionary imperative to modern treatment target. Nat Rev Immunol. (2016) 16:22–34. 10.1038/nri.2015.5 26711676 PMC5542678

[38] Mutz J Lewis CM. Lifetime depression and age-related changes in body composition, cardiovascular function, grip strength and lung function: sex-specific analyses in the UK Biobank. Aging (Albany NY). (2021) 13:17038–79. 10.18632/aging.203275 34233295 PMC8312429

[39] Dregan A Rayner L Davis KAS Bakolis I Arias de. la Torre J Das-Munshi J. Associations between depression, arterial stiffness, and metabolic syndrome among adults in the UK biobank population study: a mediation analysis. JAMA Psychiatry. (2020) 77:598–606. 10.1001/jamapsychiatry.2019.4712 31995135 PMC6990710

[40] McCaffery JM Frasure-Smith N Dubé MP Théroux P Rouleau GA Duan Q. Common genetic vulnerability to depressive symptoms and coronary artery disease: a review and development of candidate genes related to inflammation and serotonin. Psychosom Med. (2006) 68:187–200. 10.1097/01.psy.0000208630.79271.a0 16554382

[41] Park SJ Lee MG Jo M Kim G Park S. Joint effect of depression and health behaviors or conditions on incident cardiovascular diseases: a Korean population-based cohort study. J Affect Disord. (2020) 276:616–22. 10.1016/j.jad.2020.07.009 32871693

[42] Teply RM Packard KA White ND Hilleman DE DiNicolantonio JJ. Treatment of depression in patients with concomitant cardiac disease. Prog Cardiovasc Dis. (2016) 58:514–28. 10.1016/j.pcad.2015.11.003 26562328

[43] Mavrides N Nemeroff C. Treatment of depression in cardiovascular disease. Depress Anxiety. (2013) 30:328–41. 10.1002/da.22051 23293051

[44] Jha MK Qamar A Vaduganathan M Charney DS Murrough JW. Screening and management of depression in patients with cardiovascular disease: JACC state-of-the-art review. J Am Coll Cardiol. (2019) 73:1827–45. 10.1016/j.jacc.2019.01.041 30975301 PMC7871437

[45] Zambrano J Celano CM Januzzi JL Massey CN Chung WJ Millstein RA. Psychiatric and psychological interventions for depression in patients with heart disease: a scoping review. J Am Heart Assoc. (2020) 9:e018686. 10.1161/JAHA.120.018686 33164638 PMC7763728

